# J.S. Torday, N.W. Blackstone, and V.K. Rehan, *A Cell-Centered Alternative to Mainstream Evolutionary Medicine? Review of “Evidence-Based Evolutionary Medicine”*

**DOI:** 10.1093/emph/eoz010

**Published:** 2019-03-14

**Authors:** Adrian V Jaeggi

**Affiliations:** Institute of Evolutionary Medicine University of Zurich Zurich, Switzerland

This ambitious book attempts to provide a welcome evolutionary framework for complex physiology; at its core it succeeds in doing so, by illustrating the cellular–molecular mechanisms that give rise to sophisticated organ functions, their pleiotropies across organs and homologies across species, and their developmental and phylogenetic origins in response to internal and external selection pressures. The book opens with several chapters reviewing major transitions in the history of life, making a good case for multilevel selection theory to explain them. Major transitions, from eukaryotic cells to multicellular organisms to societies are selected for since larger size is a constantly open niche; yet this poses levels-of-selection conflicts and mechanisms are required to reduce variation at the lower level and increase it at the higher level. The book elaborates this for eukaryotic cells, where calcium-signaling mediates conflict among proto-mitochondria, and for multicellular organisms, where surface-to-volume constraints dictate divisions of labor among cells, whose high relatedness is ensured by returning to the unicellular zygote. The remaining chapters revolve around the evolution of cellular–molecular mechanisms involved in lipid-calcium homeostasis going back to the origins of life and the eukaryotic cell, and their iterative exaptation to produce the mineralized bones and sophisticated lung, skin, kidney and endocrine functions that characterize vertebrate physiology, all in response to major selection pressures such as increased levels of calcium in the oceans or oxygen in the atmosphere, the water–land transition, and the resulting physiological stressors. Herein the signaling pathways involved in the main authors’ study system of lung alveolar development reveal pleiotropies between organs and homologies among species, and highlight that ontogeny does indeed recapitulate phylogeny; in lung fibrosis or during physiologic stress the alveolar lipofibroblasts regress to the developmentally and phylogenetically older myofibroblasts, a process that can be reversed by intervening in the associated signaling pathway. This core story, repeated numerous times throughout the book, forms the rationale for the authors’ favored cellular–molecular view of evolution, which puts the unicellular state, the continuity of development and phylogeny, and cell-cell communication center stage (jettisoning the multilevel selection approach outlined in the first chapters).

Although its core story is important and fascinating, the book is also extremely frustrating for someone trained in neo-Darwinian evolutionary theory, which is repeatedly critiqued on shaky grounds in what oftentimes reads like a manifesto for the authors’ favored cell-centered view of evolution. Further, contra claims made on the cover this is far from a comprehensive textbook for evolutionary medicine, being highly redundant, sparsely referenced and often speculative, and lacking virtually all the classic topics that populate other evolutionary medicine texts.

The problems begin for me when the authors venture away from lung development and try to subsume topics such as the evolution of bipedalism and consciousness, ‘man’s place in the universe’, bioethics, or public health under their cellular theory of everything. For instance, they conjecture that:

‘increased efficiency of metabolism allowed for bipedalism in hominins and birds alike, freeing the forelimbs for specialization – flight, tool making, and texting. The dynamic interactions between forelimb evolution and endothermy led to higher consciousness among hominins and birds.’ (p. 186)

Such speculations come largely without citations, and ignore that the water–land transition does not inevitably lead to endothermy, as evidenced by reptiles, that endothermy does not inevitably lead to bipedalism, as evidenced by most mammals, and that ‘higher consciousness’ has little to do with any of this, as evidenced by dolphins or great apes outsmarting most birds. Such writing falls into the very teleology that the authors accuse mainstream evolutionary biology of, and is interspersed with plainly wrong facts such as humans, gorillas, and chimpanzees all descending from a common bipedal ancestor (!). Similarly, when talking about dopamine receptor alleles ‘causing’ out-of-Africa migration (p. 155), quantum theory providing a ‘radically new notion of unbroken wholeness of the entire Universe’ (p. 183), or suggesting that social systems be redesigned through an understanding of human physiology from its unicellular origins (p. 192) the authors have ventured onto increasingly thin ice indeed.

In this context, the repeated attacks on neo-Darwinian theory and manifestos for the cell-centered view of evolution are difficult to take seriously. Sidestepping active (and importantly, unresolved) debates within evolutionary biology, Williams’ antagonistic pleiotropy or Mayr’s proximate/ultimate distinction are rapidly dismissed as ‘dogmatic teleologic mechanisms and tautologic concepts’ (p. 110) and firmly replaced by Haeckel’s biogenetic law and Lamarckian inheritance. Thus, the authors claim without citing any references that epigenetic inheritance is more important than random mutation and selection, and that the phenotype is therefore merely a means of collecting epigenetic marks for the benefit of the unicellular state. Hence phantom limb sensations (chapter 13), right? It would also appear that there is ‘no experimental evidence for … evolution’ (p. 225)—contra decades of experimental evolution in labs such as Lenski’s or Stearns’—and that this alleged lack of evidence explains why ‘Creationism has held sway over evolution theory’ (p. 223). Further, only the cellular view can apparently reconcile Darwinian fitness and empathy or cooperation (p. 79), again ignoring the multilevel selection account offered initially (let alone other related work).

Ultimately, the book not only fails to do justice to evolutionary biology or comprehensively review the field of evolutionary medicine—being largely devoid of classic topics such as mismatch, life-history tradeoffs, pathogen evolution, cancer, reproductive health, nutrition, mental health etc.—but also to realize that analysis at a level higher than the cell is often more useful and predictive, especially for public health. For instance, dismissing metabolic syndrome and chronic disease as a mere ‘epiphenomenon’ (p. 94) hardly offers practical guidelines, unlike approaches developed by Gluckman, Wells, Eaton and others that are grounded in life-history theory and developmental or evolutionary mismatch. Similarly, engineering cooperative social systems requires understanding human behavior and psychology, not cellular physiology. Combined with its redundancy and repetition—virtually every chapter repeats aspects of the lung development story—frustratingly sparse referencing, far-reaching speculation, and lack of practical guidelines for research, medicine or public health, this book to me falls well short of other evolutionary medicine texts.

